# Prevalence of potentially inappropriate medications based on the STOPPFrail criteria in frail older patients with limited life expectancy: a cross-sectional study

**DOI:** 10.1186/s12877-022-03067-7

**Published:** 2022-04-27

**Authors:** Hyun-Woo Chae, Yoonhee Kim, Yewon Suh, Junghwa Lee, Eunsook Lee, Euni Lee, Jung-Yeon Choi, Kwang-il Kim, Ju-Yeun Lee

**Affiliations:** 1 Department of Pharmacy, Seoul National University Bundang Hospital, 166 Gumi-ro, Bundang-Gu, Seongnam-Si, Gyeonggi-Do 13620 Republic of Korea; 2 College of Pharmacy & Research Institute of Pharmaceutical Sciences, Seoul National University, 1 Gwanak-ro, Gwanak-gu, Seoul, 08826 Republic of Korea; 3 Department of Internal Medicine, Geriatric Centre, Seoul National University Bundang Hospital, 166 Gumi-ro, Bundang-Gu, Seongnam-Si, Gyeonggi-Do 13620 Republic of Korea; 4 Department of Internal Medicine, Seoul National University College of Medicine, 103 Daehak-ro, Jongno-gu, Seoul, 03080 Republic of Korea

**Keywords:** STOPPFrail criteria, Potentially inappropriate medication, Geriatrics, Frailty, Polypharmacy

## Abstract

**Background:**

The recently developed Screening Tool of Older Persons' Prescriptions in Frail adults with a limited life expectancy (STOPPFrail) criteria can be helpful for screening medications (PIMs), but it is yet to be widely used in clinical practice. Herein, we aimed to investigate the prevalence of PIMs based on the STOPPFrail criteria (STOPPFrail-PIM) among frail older adults with limited life expectancy admitted to the geriatric center.

**Methods:**

This was a retrospective cross-sectional study conducted in the geriatric center at an academic tertiary care hospital in Korea. We evaluated frail older adults with limited life expectancy who received comprehensive geriatric assessment (CGA) admitted between 1 January, 2019 and 30 June, 2020. Frail older adults with limited life expectancy were identified by geriatricians with retrospective records and the prevalence of STOPPFrail-PIMs was analysed by trained pharmacists. Descriptive analysis, t-test, and chi-square test were conducted using IBM SPSS software version 25.0.

**Results:**

Among 504 older adults who underwent CGA after admission, 171 frail older adults with limited life expectancy were identified by geriatricians and included in the study. An average of 11.3 ± 4.7 medications were administered regularly to each patient before admission. Overall, 97.1% (166/171) had at least one STOPPFrail-PIM, and the mean number of STOPPFrail-PIM was 4.2 ± 2.8. Drugs without clear clinical indication (A2) were the most frequent pre-admission STOPPFrail-PIM, followed by lipid-lowering therapies (B1) and neuroleptic antipsychotics (D1). The number of STOPPFrail-PIM was significantly lower at discharge than that at admission, with the decrease being the highest for A2 at 94.7%.

**Conclusions:**

Most frail older adults with limited life expectancy had at least one STOPPFrail-PIM at admission, and the rate of STOPPFrail-PIM decreased significantly at discharge after the geriatric multidisciplinary team care. Further studies are needed to investigate the association between the use of STOPPFrail-PIM and adverse consequences in frail older adults.

## Background

Many older adults have more than one underlying disease, and the number of comorbidities increases with ageing [1]. One study identified that 40% of people aged ≥ 65 years take five or more medications, and 12% take more than 10 medications [2]. The risk of adverse drug reactions increases with the number of medications; thus, older adults on polypharmacy, especially frail older adults, are vulnerable to adverse drug reactions [3, 4]. Particularly, older adults living in facilities such as nursing homes or long-term care hospitals have poor survival outcomes because they become frailer and have multiple comorbidities and medications [5, 6].

When life expectancy is limited, the quality of life is prioritised over strict control of chronic diseases or long-term preventative therapy. This is because it is challenging to evaluate the long-term drug benefit in patients with limited life expectancy [7]. Additionally, polypharmacy is associated with worse quality of life in patients approaching the end of life [8]. Therefore, futile medications should be deprescribed in frail older patients.

The Screening Tool of Older Persons' Prescriptions in Frail adults with a limited life expectancy (STOPPFrail) criteria is a 27-section screening tool developed for identifying potentially inappropriate prescriptions in frail older adults with limited life expectancy [9]. The criteria are applicable to frail patients with a life expectancy of less than a year and those with cognitive or physical impairment. Additionally, symptom control should be prioritised over its prevention [10]. However, the STOPPFrail criteria are yet to be widely used, and a few studies have analysed the prevalence of potentially inappropriate medications according to the STOPPFrail criteria (STOPPFrail-PIMs) mostly in Europe. However, few studies have evaluated the prevalence of PIM use in frail older adults based on STOPPFrail criteria among Asian countries including Korea.

We aimed to estimate the prevalence of STOPPFrail-PIMs among the pre-admission and discharge medications of frail older adults with limited life expectancy admitted to the geriatric center in a tertiary care academic medical center.

## Methods

### Study design and setting

This was a retrospective cross-sectional study of frail older adults (aged at least 65 years) with limited life expectancy who received comprehensive geriatric assessment (CGA) after admission to the geriatric center of Seoul National University Bundang Hospital between 1 January, 2019 and 30 June, 2020.

The geriatric center of the study hospital has a multidisciplinary team of doctors, pharmacists, nurses, and nutritionists to conduct a multicomponent intervention based on comprehensive geriatric assessment (CGA). As a part of CGA, clinical pharmacists perform medication review and recommend identifying drug related problems including drugs without indications, duplications, inappropriate dose, and interactions as well as potentially inappropriate medications (PIMs) based on Beers criteria [11].

The minimum sample size of 124 was determined to estimate the prevalence of STOPPFrail-PIMs using a single population proportion assuming 91.2% prevalence from Lavan et al. [12] with 95% confidence and 5% margin of error.

This study was approved by the relevant institutional review board of Seoul National University Bundang Hospital (IRB number B-2008/633–104). The need for informed consent was waived by Seoul National University Bundang Hospital institutional review board owing to the retrospective nature of the study.

### Participants and data collection

The STOPPFrail criteria are eligible for frail patients with a life expectancy of less than one year and for those with cognitive or physical impairment among patients with no possibility of fundamental recovery. Additionally, symptom control should be prioritised over its prevention. To identify frail older patients with limited life expectancy, we screened patients with severe cognitive impairment (Korean version of the Mini-Mental State Examination score < 10) or severe to complete dependence in activities of daily living (ADLs) (Modified Barthel Index score < 50) for severely cognitive or functionally impaired patients, then checked the living condition to understand whether the patients need symptom control over its prevention. We considered symptom control was prioritised for patients transferred from facilities (nursing homes or long-term care hospitals). Among those that met the criteria above, geriatricians finally selected frail older adults with limited life expectancy by reviewing the electronic medical records of patients. Patients who were not administered any medication at admission or who died during hospitalisation were excluded. When patients were admitted more than once during the study period, only the first admission records were included.

The following data were collected from the electronic health records: age, sex, living condition, and list of medications upon admission and prescription at discharge. Additionally, we collected CGA data including ADLs, Lawton-Brody instrumental activities of daily living, Korean version of the Mini-Mental State Examination, Mini Nutritional Assessment, and Charlson Comorbidity Index [13].

The medication use of each patient was analysed by trained pharmacists not involved in the care of patients included in the study. The number of medications was determined by the active ingredients and not by the number of pills, i.e., the number of active ingredients in fixed dose combinations was counted. Topical agents and local action drugs except for inhalers were excluded. Polypharmacy and excessive polypharmacy were defined as the use of ≥ 5 and ≥ 10 medications, respectively [14]. The prevalence of potentially inappropriate medications (PIM) was evaluated based on the STOPPFrail criteria (STOPPFrail-PIMs) and the Beers criteria 2019 (Beers-PIM), which is a widely used tool to identify PIMs in older adults [11]. We investigated the prevalence of five parts of Beers-PIM to understand the number of PIMs that can be estimated by the tool not targeting frail older adults as reference. As needed drugs recorded in the chart were not included when estimating the number of regular medications and PIMs.

As previous studies suggested that prevalence and frequently used PIMs vary according to the socio-demographic factors [15–17], commonly used STOPPFrail-PIMs by age group, sex, and living conditions were determined.

### Statistical analysis

Continuous variables were described as means and standard deviations, while categorical variables were reported as percentages. A paired t-test was used to compare the total number of drugs or STOPPFrail-PIM between admission and discharge. Student’s t-test and the chi-square test were used to analyse STOPPFrail-PIM between populations. All statistical analyses were performed using IBM SPSS software version 25.0 (IBM Korea, Seoul), and significance was tested at α = 0.05 and 95% confidence interval.

## Results

### Baseline patient characteristics

**Fig. 1:**
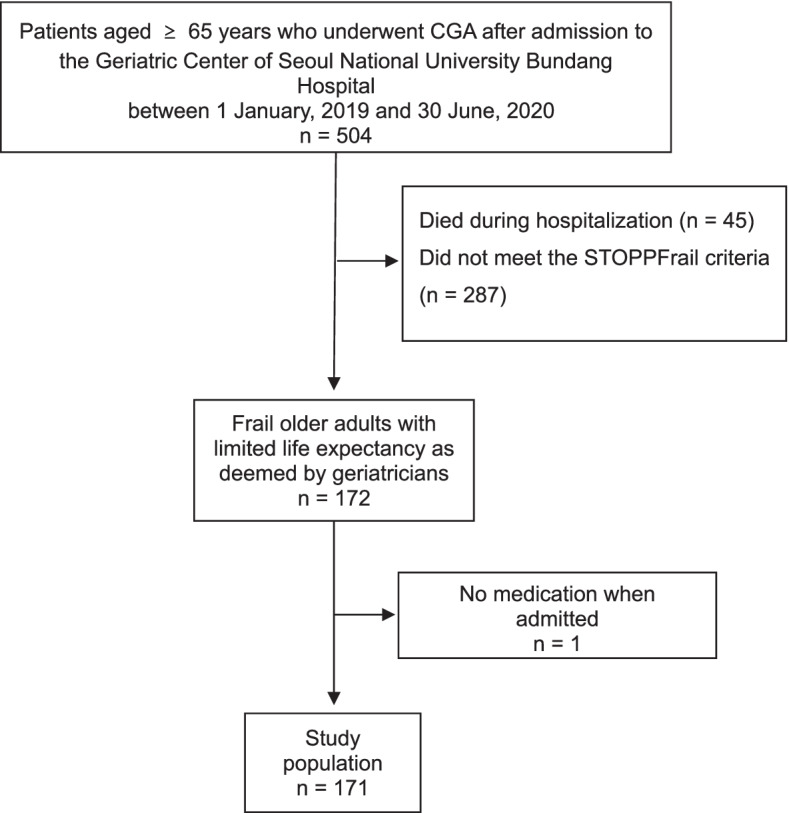
Patient inclusion flowchart. CGA: comprehensive geriatric assessment

Among the 504 eligible patients, 172 patients were classified as frail older adults with a limited life expectancy. After excluding one patient without medication at the time of admission, 171 patients were finally included. Patient inclusion flowchart is presented in Fig. 1. The baseline characteristics of the study population are shown in Table 1. The mean age of the study population was 83.1 ± 7.5 years, and females accounted for a slightly higher proportion (53.4%, *n* = 93) of the group than male. Patients from long-term care hospitals and nursing homes accounted for 59.6% (*n* = 102) and 40.4% (*n* = 69) of the total population, respectively (Table 1).

**Table 1:** Baseline patient characteristics (*n* = 171)

Variables	
Sex	
Female	92 (53.8%)
Male	79 (46.2%)
Age, year	83.1 ± 7.5
< 85	105 (61.4%)
≥ 85	66 (38.6%)
Height, cm	159.8 ± 8.9
Weight, kg	51.0 ± 11.4
ADL	7.2 ± 17.6
Independent ADL (100)	0 (0%)
Minimal dependent ADL (91–99)	3 (1.8%)
Mild dependent ADL (75–90)	0 (0%)
Moderate dependent ADL (50–74)	4 (2.3%)
Severe dependent ADL (25–49)	9 (5.3%)
Full dependent ADL (0–24)	155 (90.6%)
IADL	
Independent IADL	0 (0%)
Dependent IADL	171 (100%)
MMSE-KC	1.4 ± 3.2
MNA	11.6 ± 4.7
Charlson Comorbidity Index	2.8 ± 1.8
Nu-DESc	2.1 ± 0.7
Living condition	
Nursing home	69 (40.4%)
Long-term are hospital	102 (59.6%)

Data were presented as mean ± standard deviation or N (%)

*ADL* Activities of Daily Living, *IADL* Instrumental Activities of Daily Living, *MMSE* Mini Mental State Examination, *MNA* Mini Nutritional Assessment, *Nu-DESc* Nursing Delirium Scale

### Medication use and prevalence of STOPPFrail-PIM at admission and discharge

Before admission, each patient was taking an average of 11.3 ± 4.7 regular medications. Of the 171 patients, 165 patients (96.5%) were taking five or more medications. There were 100 (58.5%) patients taking 10 or more regular medications (excessive polypharmacy). On discharge, each patient was taking 7.9 ± 3.5 regular medications on average, and the rate significantly reduced compared to that at pre-admission (*p* < 0.001). The number of non-polypharmacy (< 5 medications) patients increased to 29 (17%), while the number of excessive polypharmacy patients decreased to 52 (30.4%).

Frail older adults used more STOPPFrail-PIM than Beers-PIM. Most patients (96.1%) used one or more STOPPFrail-PIM before admission, at an average of 4.2 ± 2.8. The mean number of STOPPFrail-PIM at discharge significantly decreased to 1.4 ± 1.3 per patient (*p* < 0.001). Meanwhile, Beers-PIM was relatively lower at a mean of 1.3 ± 1.2 medications pre-admission, and this was significantly decreased to 0.6 ± 0.8 at discharge (*p* < 0.001) (Table 2).

**Table 2:** Comparison of medication use at admission and at discharge

	**Admission**	**Discharge**	***p*-value**
**Regular medications**	11.3 ± 4.7	7.9 ± 3.5	< 0.001
< 5	6 (3.5%)	29 (17.0%)	
5–9	65 (38.0%)	90 (52.6%)	
10–14	64 (37.4%)	41 (24.0%)	
15–19	26 (15.2%)	11 (6.4%)	
≥ 20	10 (5.8%)	0 (0%)	
**Beers-PIM**	1.3 ± 1.2	0.6 ± 0.8	< 0.001
0	49 (28.7%)	103 (60.2%)	
1–2	95 (55.6%)	63 (36.8%)	
3–4	26 (15.2%)	5 (2.9%)	
≥ 5	1 (0.6%)	0	
**STOPPFrail-PIM**	4.2 ± 2.8	1.4 ± 1.3	< 0.001
0	5 (2.9%)	43 (25.1%)	
1–2	46 (26.9%)	94 (55.0%)	
3–4	55 (32.2%)	31 (18.1%)	
5–6	35 (20.5%)	3 (1.8%)	
7–8	20 (11.7%)	0	
≥ 9	10 (5.8%)	0	

Data were presented as mean ± standard deviation or N (%)

*STOPPFrail-PIM* potentially inappropriate medication according to STOPPFrail (Screening Tool of Older Persons’ Prescriptions in Frail adults with a limited life expectancy) criteria

*Beers-PIM* potentially inappropriate medication according to Beers Criteria (2019)

### Frequent type of STOPPFrail-PIMs and comparison between admission and discharge

Overall, 714 of the 1,953 (36.6%) medications before admission were classified as STOPPFrail-PIM, and it significantly decreased to 245 of 1,360 (18.0%) at discharge (*p* < 0.001). From section A1 to J3, drugs without clear clinical indication (A2) were the most frequent pre-admission STOPPFrail-PIM, accounting for 413 of the 1953 medications (130 of 171 patients). This was followed by lipid-lowering drugs (B1), neuroleptic antipsychotics (D1), and anti-platelet agents (C1). At discharge, B1 was the most frequently prescribed medication (*n* = 58) followed by diabetic oral agents (I1) (*n* = 35) and D1 (*n* = 32). The proportion of A2, i.e., the most frequent STOPPFrail-PIM before admission, significantly decreased to 22 at discharge. Meanwhile, I1 increased from 27 instances at pre-discharge to 35 at discharge (Table 3).

**Table 3:** Common types of STOPPFrail-PIM at pre-admission and at discharge

**STOPPFrail Criteria Section**		**Instance (n)**		**Reduction rate (%)**	***p*-value**
		**Admission**	**Discharge**		
A2	Any drug without clear clinical indication	413	22	94.7	< 0.001
B1	Lipid lowering therapies	66	58	12.1	0.088
D1	Neuroleptic antipsychotics	50	32	36.0	0.001
C1	Anti-platelet for primary prevention	41	19	53.7	< 0.001
I1	Diabetic oral agents for stringent control	27	35	-29.6	0.131
G1	Calcium supplementation	21	18	14.3	0.468
D2	Memantine without BPSD	21	16	23.8	0.025
J1	Multivitamins	17	13	23.5	0.395
E2	High-dose H2RA (full therapeutic dose with ≥ 8 weeks)	13	4	69.2	0.012
G4	Long-term oral NSAIDs (≥ 2 months)	13	2	84.6	0.001
E1	High-dose PPI (full therapeutic dose with ≥ 8 weeks)	9	11	-22.2	0.594
H2	Alpha blockers with urinary bladder catherization	5	5	0	1.000
G2	Anti-resorptive/bone anabolic drugs for osteoporosis	4	1	75.0	0.083
E3	Gastrointestinal antispasmodics	3	1	66.7	0.158
G5	Long-term oral steroids (≥ 2 months)	3	4	-33.3	0.565
F1	Theophylline use other than asthma	2	1	50.0	0.319
J2	Nutritional supplements	2	3	-50.0	0.707
A1	Any drug that patient persistently fails to take or tolerate	1	0	100	0.319
B2	Alpha-blockers for hypertension	1	0	100	0.319
F2	Leukotriene antagonists	1	0	100	0.319
G3	SERMs for osteoporosis	1	0	100	0.319
H1	5-Alpha reductase inhibitors with urinary bladder catherization	0	0		
H3	Muscarinic antagonist with urinary bladder catherization	0	0		
I2	ACE-inhibitors for diabetic nephropathy	0	0		
I3	Angiotensin receptor blockers for diabetic nephropathy	0	0		
I4	Systemic estrogens for menopausal symptoms	0	0		
J3	Prophylactic antibiotics	0	0		
**Total**		**714**	**245**	**65.7**	< 0.001

STOPPFrail-PIM: potentially inappropriate medication according to STOPPFrail (Screening Tool of Older Persons’ Prescriptions in Frail adults with a limited life expectancy) criteria

BPSD, behavioral and psychiatric symptoms of dementia; H2RA, H2 receptor antagonist; NSAID, non-steroidal anti-inflammatory drug; PPI, proton pump inhibitor; SERM, selective estrogen receptor modulator; ACE, angiotensin converting enzyme

We compared the use of STOPPFrail-PIM by sex, age, and living condition and investigated whether these factors influenced the prevalence of STOPPFrail-PIM. The frequent sections of the STOPPFrail criteria were almost identical in all groups. The four most frequent sections in sex and age groups were A2, B1, D1, and C1. Meanwhile, the fifth most frequent section varied. Considering sex, the fifth most common STOPPFrail-PIM was calcium supplementation (G1) in females, whereas it was I1 in males. Regarding age, the fifth most common STOPPFrail-PIM was I1 in patients aged under 85 years, whereas it was memantine (D2) and G1 among those who aged ≥ 85 years. Considering the living condition, patients living in nursing homes were likely to take D2 as the fifth most frequent STOPPFrail-PIM, whereas the fourth and fifth STOPPFrail-PIM were I1 and C1, respectively among patients living in long-term care hospitals.

Notably, we found that the living condition could influence STOPPFrail-PIM use. Patients living in nursing homes were more likely to take B1 (49.3%, *p* = 0.004), C1 (31.9%, *p* = 0.007), and long-term oral NSAIDs (G4) (14.5%, *p* = 0.005) than those living in long-term care hospitals. Meanwhile, sex and age had no significant influence on STOPPFrail-PIM use (Table 4).

**Table 4:** Comparison of frequently used STOPPFrail-PIM by sex, age, and living condition

STOPPFrail Criteria Section		Sex					Age					Living Condition				
		**Female** **(*n* = 92)**		**Male** **(*n* = 79)**		***p*-value**	** < 85 years** **(*n* = 105)**		** ≥ 85 years** **(*n* = 66)**		***p*-value**	**Nursing home** **(*n* = 69)**		**Long-term care hospital** **(*n* = 102)**		***p*-value**
		**n**	**%**	**n**	**n**		**n**	**%**	**n**	**%**		**n**	**%**	**n**	**%**	
A2	Any drug without clear clinical indication	65	70.6	65	82.3	0.76	82	78.1	48	72.7	0.423	50	72.5	80	78.4	0.37
B1	Lipid lowering therapies	32	34.8	30	38.0	0.665	40	38.1	22	33.3	0.528	34	49.3	28	27.5	0.004
D1	Neuroleptic antipsychotics	22	23.9	19	24.1	0.983	25	23.8	16	24.2	0.949	18	26.1	23	22.5	0.595
C1	Antiplatelet for primary prevention	19	20.7	18	22.8	0.736	23	21.9	14	21.2	0.915	22	31.9	15	14.7	0.007
I1	Diabetic oral agents for stringent control	11	12.0	14	17.7	0.453	19	18.1	6	9.1	0.105	9	13.0	16	15.7	0.631
G1	Calcium supplementation	14	15.2	7	8.9	0.207	13	12.4	8	12.1	0.960	9	13.0	12	11.8	0.803
D2	Memantine without BPSD	10	10.9	11	14.0	0.544	13	12.4	8	12.1	0.960	12	17.4	9	8.8	0.094
J1	Multivitamins	7	7.6	6	8.0	0.287	9	8.6	4	6.1	0.105	0	0	13	12.7	-
E2	High-dose H2RA (full therapeutic dose with ≥ 8 weeks)	8	8.7	5	6.3	0.560	8	7.6	5	7.6	0.992	2	2.9	11	10.8	0.056
G4	Long-term oral NSAIDs (≥ 2 months)	7	7.6	6	7.6	0.991	8	7.6	5	7.6	0.992	10	14.5	3	2.9	0.005

*STOPPFrail-PIM* potentially inappropriate medication according to STOPPFrail (Screening Tool of Older Persons’ Prescriptions in Frail adults with a limited life expectancy) criteria

*H2RA* H2 receptor antagonist, *NSAID* non-steroidal anti-inflammatory drug, *PPI* proton pump inhibitor

## Discussion

Although the STOPPFrail criteria can be helpful for identifying PIMs in frail older adults with limited life expectancy, it is yet to be widely used in practice. In this study, most of the subjects used one or more STOPPFrail-PIM before hospitalisation, indicating a high rate of PIM use among frail older adults with limited life expectancy. Overall, 97.1% (166/171) of the patients took at least one STOPPFrail-PIM at admission, and 36.6% of all medications (714/1953) were STOPPFrail-PIMs.

Lavan et al. evaluated frail older adults with poor one-year survival prognosis and identified PIM usage among 91.2% of patients, with 37.7% of the prescribed medications being STOPPFrail-PIMs [12]. Curtin et al. also reported that 90.8% of frail older patients used STOPPFrail-PIM, with a mean of 2.4 ± 1.4 STOPPFrail-PIM per patient [7]. Our findings are consistent with those of two previous studies, supporting that most frail older adults use PIMs according to the STOPPFrail criteria. Another study by Curtin et al. also found that 81.5% of the patients were prescribed at least one STOPPFrail-PIM at discharge [18]. The three most prevalent STOPPFrail-PIMs in our study population were drugs without clear clinical indication, lipid-lowering drugs, and neuroleptic antipsychotics. This was consistent with the findings of previous studies by Lavan [12] and Curtin [7] where drugs without clear clinical indication was the most prevalent STOPPFrail-PIMs.

The prevalence of patients prescribed at least one STOPPFrail-PIM at discharge significantly decreased to 74.9% after CGA although we did not adopt the STOPPFrail criteria for the medication review. This could be because the multidisciplinary team including pharmacists who performed medication re views regarding indication, effectiveness, safety, and adherence and identified PIMs. This finding was consistent with that of a post-analysis study conducted in Belgium [19] wherein a significant reduction in STOPPFrail-PIM use was found when clinical pharmacists intervened on PIMs based on the STOPP/START and Beers criteria. 

However, the frequently used STOPPFrail-PIMs at baseline differed from those at discharge. In Fournier’s study, proton pump inhibitors, calcium supplementation, and anti-platelet agents were the most frequent STOPPFrail-PIMs at baseline. However, after an 8-month follow-up, calcium supplementation and diabetic oral agents became the most frequent PIMs in the intervention group. In the current study, the prevalent STOPPFrail-PIMs at admission were drugs without clear clinical indication and anti-platelet agents, whereas they were lipid-lowering therapies and diabetic oral agents at discharge. This overall difference may be due to variations in the study population, number of criteria used, and healthcare settings as well as the data source. In Fournie r’s study, six criteria including ‘drugs without clear clinical indication’ (the most prevalent PIM in this study) were excluded [19]. Specifically, the prevalence of high-dose proton pump inhibitor (PPI) (full therapeutic dose with ≥ 8 weeks) (5.2%) in this study was lower than that observed in Fournier’s study (18.0%) although the definition of duration and dose were same. This finding could be partly explained by the fact that PPIs are available only by prescription and the reimbursement restriction on PPI use other than approved indications in Korea. The prevalence of PPI use at admission without considering dose and duration was 29.2%, but the PPI was used at less than full-strength dose in most cases (21.6%). From this finding we can infer that long-term use of maintenance-dose PPI is more common than that of full therapeutic dose PPI in Korea.

Considering the influence of type of living conditions on PIMs, the patients transferred from nursing homes were more likely to take B1, C1, and G4 STOPPFrail-PIM than those transferred from long-term care hospitals. This could be possibly attributed to the difference in prescription system and patient accessibility between nursing homes and long-term care hospitals. In Korea, long-term care hospital services and nursing home services are covered by the National Health Insurance and Long-Term Care Insurance for the Elderly, respectively. In long-term care hospitals, physicians are available 24 h a day and can prescribe medications; however, pharmacists provide 16-h weekly service mainly spending time on filling prescriptions and they mostly cannot provide clinical medication review services [20]. In nursing homes, contracted physicians only visit patients as per a certain schedule (usually twice a month) for clinical examination, ordering nursing treatments, or hospital referrals. There are no regulatory requirements regarding medication management in Korea unlike in other countries with enacted laws to optimize drug management in long-term care facilities [21]. Doctors are available every day; thus, they are responsible for deprescribing anti-platelets or NSAIDs in long-term care hospitals, whereas, in nursing homes, prescriptions are refilled without evaluating patients’ clinical conditions as often as they are evaluated in long-term care hospitals.

We also found a 65% reduction of STOPPFrail-PIM at the time of discharge compared to that at admission. Although pharmacists from the multidisciplinary team intervened in the patients’ medications only on the basis of the Beers criteria, PIMs listed in the STOPPFrail criteria were also deprescribed spontaneously. Drugs without clear clinical indication, which were the most prevalent STOPPFrail-PIMs at admission and were highly reduced at discharge, fall under the general medication review process and are not specific to the STOPPFrail criteria.

### Strengths and limitations

In this study, we analysed the medications that frail older adults with limited life expectancy have taken at nursing facilities before admission. The results showed that a large number of STOPPFrail-PIMs were used. This supports the need for medication management based on the STOPPFrail criteria in frail older adults with limited life expectancy living in nursing facilities.

To the best of our knowledge, this was the first study in Korea that provided a better understanding of medication use in frail older adults with limited life expectancy based on the STOPPFrail criteria in Asian countries; it increased the generalizability of STOPPFrail criteria, irrespective of the difference in medication use or healthcare system. The distinction among the countries will provide insight for developing better tools for the clinical use of PIM criteria among frail older adults. However, this study has some limitations. First, our findings may have limited generalisability because this research was conducted at a single institution. Nevertheless, the findings indirectly reflect the status of several nursing institutions because we analysed pre-admission medications of patients transferred from various nursing facilities. Second, as limited life expectancy was defined by geriatricians based on a retrospective review of medical records, the complete clinical condition of the patients might not be considered, possibly leading to inaccurate life expectancies. However, a number of similar previous studies were also conducted retrospectively using medical records. Third, variables such as the time duration of patients in nursing facilities or detailed medical history was not available from the medical records; thus, we could not analyse the predictive factors associated with the use of STOPPFrail-PIM; this would have enabled targeting the population at risk. Additionally, it seems necessary to study the relation between the use of STOPPFrail-PIM and the prognosis of the patients [22], and the effect of real-life application of the STOPPFrail criteria on the deprescription of STOPPFrail-PIM in frail older adults with limited life expectancy needs to be confirmed in further studies. Also studies evaluating the impact of medication review based on STOPPFrail criteria prospectively as part of clinical practice are warranted.

## Conclusion

The majority of frail older adults with limited life expectancy transferred from nursing facilities were administered at least one STOPPFrail-PIM. The most frequent STOPPFrail-PIMs were any drugs without clear clinical indications, lipid-lowering therapies, neuroleptic antipsychotics, and anti-platelets. Therefore, it seems necessary to refer to the STOPPFrail criteria while reviewing medications for frail older adults with limited life expectancy. Further studies are needed to investigate the association between the use of STOPPFrail-PIM and adverse consequences in frail older adults. 

## Data Availability

The datasets used and/or analysed during the current study are available from the corresponding authors on reasonable request.

## Abbreviations

STOPPFrail: Screening tool of older persons’ prescriptions if frail adults with a limited life expectancy
PIM: Potentially inappropriate medication
STOPPFrail-PIM: PIMs according to STOPPFrail criteria
Beers-PIM: Potentially inappropriate medication according to Beers criteria
CGA: Comprehensive geriatric assessment
ADL: Activities of daily living
IADL: Instrumental activities of daily living
MMSE: Mini mental state examination
MNA: Mini nutritional assessment
Nu-DESc: Nursing delirium scale
BPSD: Behavioral and psychiatric symptoms of dementia
H2RA: H2 receptor antagonist
NSAID: Non-steroidal anti-inflammatory drug
PPI: Proton pump inhibitor
SERM: Selective estrogen receptor modulator
ACE: Angiotensin converting enzyme
